# Medical Journal Advertising

**Published:** 1911-05

**Authors:** 


### Medical Journal Advertising.

Since advertising in medical journals is the principal means the members of this association have in bringing their work to the notice of the profession and since it is unsatisfactory and unfair for men who have won an honorable place in the profession by conscientious and diligent study and by honorable and fair dealing to be brought into competition, on terms of equality, with irregular and disreputable institutions, as is done when the advertisements of such disreputable institutions appear in the pages of the same journals containing the cards of institutions of good standing, therefore the members of this association pledge themselves not to run an advertisement in any medical journal or medical directory which admits to its pages the advertisements of irregular institutions.

Any institution or physician guilty of any of the following acts shall be deemed irregular for the purposes of this organization:

1. Use of a secret remedy or secret form of treatment.
2. Publishing testimonials of cures.
3. Guaranteeing cures.
4. Sending out prepared or home treatments for drug addictions.
5. Paying commissions to physicians for sending patients.
6. Advertising in the lay press in any manner except the insertion of a simple card giving the name and location of the institution or physician, class of patients treated, office hours of the superintendent or medical director and telephone number; provided, however, that the publication of such cards shall be confined to the lay press of the county in which the institution or physician is located.

[Explanatory Note 1.—The reason for each of the provisions above given would seem sufficiently clear, except the fourth. To those who have not given attention to such matters it might seem that to prohibit the sending out of home treatments for the drug addictions would be an unreasonable restriction and one that would interfere with the natural rights of individuals and of physicians, but that is not the purpose of the rule. The reason for such restrictions is this: In almost every instance remedies advertised as home cures for the morphine and other drug addictions are merely some concealed form of opiate. It is not difficult to switch the drug user from his morphine or other opiate on to the "cure," which he is assured is merely a harmless tonic. This assurance he accepts as true and under the belief that he has been freed from his drug slavery and in the buoyancy and hope that such a deliverance would bring, he writes glowing testimonials of his cure while still under the influence of the supposedly harmless tonic. When he attempts to leave off this tonic, he finds that he is totally unable to do so; in fact, that he is as much a slave to the "cure" as he was to his original drug, and that now, instead of being able to buy his drug supply in the open market at a reasonable price, he is compelled to send ten or more dollars per month to his home-cure vendor for his drug supply, as he can not readily leave it off and resume his former drug. These "cures" usually contain a combination of narcotics, by the use of which the victim acquires a double or triple addiction instead of the single one which he had before the use of such "cure." These home treatments do not really cure the morphine or other drug addiction. This fact is known to their vendors as well as to us; therefore, we are compelled to conclude that one who continues to advertise and send out such a "cure" does so with fraudulent intent, and is, therefore, unworthy of association with gentlemen, and should not be allowed to practice his deception on an unsuspecting public by using space in the columns of medical journals.]

Note 2.—The above Constitution has been agreed upon by correspondence between the medical directors of a number of institutions as the basis of a temporary organization to stand until the annual meeting to be held in connection with the session of the American Medical Association in June. It will come up for confirmation or amendment at that meeting. Owners or superintendents of institutes, who are in accord with the objects of this organization, are requested to send their applications for membership to the Secretary.—Extract from the Constitution of the American Association of Medical Journal Advertisers, S. G. Burnett, President; G. H. Moody, Vice-President; Geo. S. Pettey, Secretary

---

Lavage of the stomach preparatory to an operation for intestinal obstruction had best be done before anesthetizing. Performed during narcosis the procedure *may* cause alarming embarrassment of respiration and, if the throat *should* become flooded with mucous or stomach content, as occasionally happens, an aspiration pneumonia is very apt to follow.—*American Journal of Surgery.*

